# The feasibility and safety of eustachian tube dilation with a standard endovascular balloon: a clinical pilot study

**DOI:** 10.1186/s40463-022-00599-1

**Published:** 2023-02-28

**Authors:** Valerie Dahm, Justin T. Lui, Soyeon Jung, Vincent Y. Lin, Joseph M. Chen, Trung N. Le

**Affiliations:** 1 Department of Otolaryngology – Head and Neck Surgery, Sunnybrook Research Institute, Toronto, Canada; 2 Department of Otolaryngology – Head and Neck Surgery, University of Toronto, Toronto, Canada; 3 Sunnybrook Health Sciences Centre, Toronto, Canada

**Keywords:** Eustachian tube dysfunction, Eustachian tube dilation, Balloon dilation of the eustachian tube, Endoscopy, Endovascular balloon

## Abstract

**Background:**

Obstructive eustachian tube dysfunction is a common pathology, generally accepted as the underlying cause for chronic ear diseases. Eustachian tube dilation has shown promising results in randomized controlled trials, but is a costly procedure.

The aim of the current study was to assess the feasibility of transnasal balloon dilatation of the eustachian tube with an endovascular balloon in the context of ease of use, maneuverability, and safety.

**Methods:**

Clinical pilot study carried out at a university tertiary care facility. In total, twelve patients, were included over a period of 6 months. All patients underwent tympanoplasty or tympanomastoidectomy surgeries. Eustachian tube dilation was carried out transnasal using an endovascular balloon. A computed tomography was carried out after surgery to assess for any potential damages and compared to preoperative imaging studies. Postoperative endoscopy was performed intraoperatively and at follow up to assess for any potential damages.

**Results:**

All eustachian tube dilations were carried out successfully. No severe adverse events were noted during the procedure, in the postoperative period, or on imaging studies. Minor adverse events such as mild intraoperative bleeding was managed in a routine fashion.

**Conclusions:**

Balloon dilation of the eustachian tube with the endovascular balloon was feasible and safe in all cases. It is likely a more cost-effective alternative to commercially available balloons with similar designs and specifications.

*Trial registration* The study was registered at clinicaltrials.gov; NCT04809753, initial release February 24th, 2021.

**Graphical Abstract:**

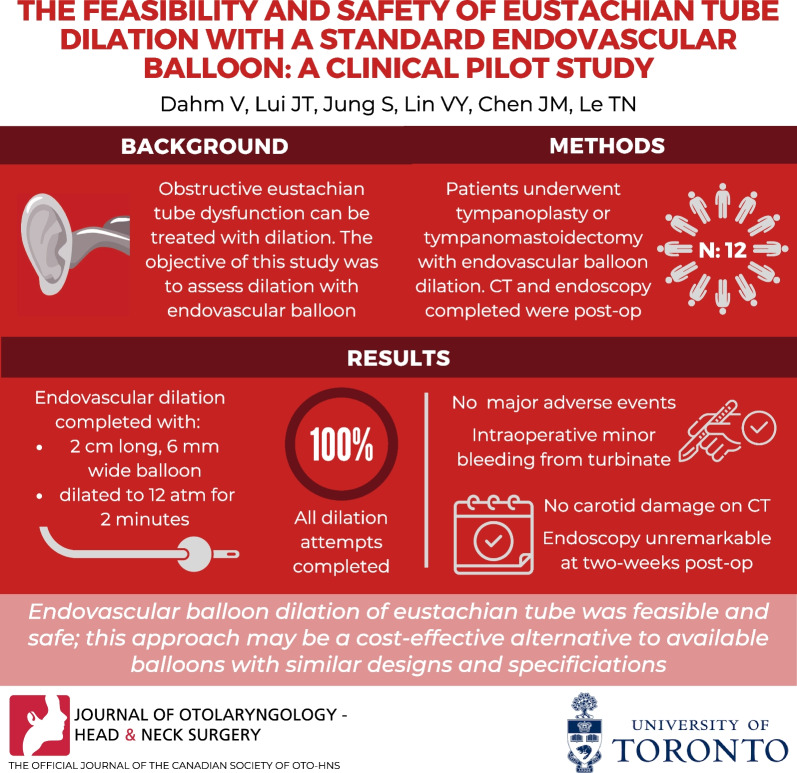

## Background

Obstructive eustachian tube dysfunction (OETD) is a common affliction, generally accepted as the underlying cause for chronic ear diseases, such as chronic otitis media with effusion [1]. It is associated with symptoms such as aural fullness, otalgia, inability to perform a Valsalva maneuver, and hearing loss [2]. Medical treatment of OETD based on nasal steroids, decongestants, and antihistamines are commonly prescribed to reduce symptoms with little evidence of benefit [3–6]. Direct manipulation of the eustachian tube (ET) was attributed to Adam Politzer in the late nineteenth century that involved catheterization under local anesthesia with insufflation and topical therapy, often referred to as “Politzerization”, and “inflating the drum-head” [7]. The lack of benefit and the invasive nature of this treatment led to its gradual demise. In modern times, the development of endoscopic sinus surgery and the popularity of balloon sinoplasty naturally led to the adoption of balloon dilation of the eustachian tube (BDET). By inserting and inflating a non-compressible balloon within the lumen of the ET, a single treatment can potentially lead to a sustained improvement of ET function [8]. This technique was first investigated in cadavers [8, 9], followed by clinical pilot trials in 2010–2011 [10, 11]. A subsequent randomized controlled trial by Poe et al., which compared medical management alone with medical management in combination with BDET, showed that there was a significant advantage for the patients in the surgical treatment arm—normalization of tympanogram at 6 weeks after surgery in 51.8% (72/139) versus 13.9% (10/72) patients [12]. The U.S. Food and Drug Administration (FDA) has issued a Class II approval for two eustachian dilation balloons for OETD. These single-use balloon devices are generally priced at over $US1000 per unit (personal communication with manufacturer). McCoul et al. reported an average cost of USD$6072 per person for the entire procedure of BDET in a cross-sectional U.S. national health care database study [13].

When considering the history of endoluminal dilatation, the use of inflatable balloon is pervasive in gastrointestinal, vascular and cardiac diseases. The design and specifications of the endovascular balloons, routinely used for percutaneous transluminal angioplasty, have similar size and pressure properties as the BDET device. The purchasing unit cost for an endovascular balloon (EVB) including the inflation device is USD$170 (personal communication with manufacturer). Such EVB is approved for clinical use world-wide. A pre-clinical feasibility study in a cadaveric model was performed and showed comparable dilation volumes of the ET using the EVB with no structural damage or other safety concerns [14]. The objective of the presented study was to assess the feasibility of balloon dilatation of the eustachian tube with an endovascular balloon in the context of ease of use, maneuverability, and safety in twelve patients.

## Methods

### Participants

In total, twelve patients were included between February 24th and August 15th, 2021, details are listed in Table 1. Patients, who were scheduled to undergo tympanoplasty or tympanomastoidectomy with symptoms and signs of OETD were asked to participate in the study.

**Table 1:** Patient details and results of ETDQ-7 questionnaires, tympanogram (tymp.), average conductive hearing loss (CHL) and pure tone average (4-PTA) before (pre) and 2-months after (post) surgery

		Sex	Age	Side	ETDQ pre	ETDQ post	Tymp. pre	Tymp. post	CHL pre	CHL post	4PTA pre	4PTA post
1	TM Perforation	F	61	R	28	18	B	A	20 dB	19 dB	48 dB	34 dB
2	CSOM	F	24	R	12	16	A	A	50 dB	8 dB	69 dB	23 dB
3	CSOM	M	42	L	41	39	DNT	B	6 dB	23 dB	13 dB	42.5 dB
4	Cholesteatoma	F	40	L	29	7	C	C	13 dB	19 dB	28 dB	28 dB
5	Cholesteatoma	F	30	L	7	9	B	B	29 dB	29	45 dB	45 dB
6	Cholesteatoma	F	66	R	9	9	A	A	33 dB	50 dB	74 dB	> 100 dB
7	Cholesteatoma	F	53	L	17	16	B	B	29 dB	41 dB	36 dB	60 dB
8	Cholesteatoma	F	77	R	40	DNT	B	B	CNT	CNT	> 100 dB	> 100 dB
9	TM Perforation	F	28	L	39	14	B	A	20 dB	5 dB	26 dB	25 dB
10	Cholesteatoma	M	34	L	38	29	DNT	DNT	34 dB	20 dB	51 dB	34 dB
11	TM Perforation	F	24	L	34	28	CNS	A	6 dB	0 dB	23 dB	18 dB
12	Cholesteatoma	F	25	R	30	8	C	C	31 dB	39 dB	46 dB	58 dB

*DNT* Did not test, *CNS* Could not seal, *CNT* Could not test, *dB* Decibel. *A* Type A tympanogram, *B* Type B tympanogram, *C* Type C tympanogram. *F* Female, *M* Male, *L* Left, *R* Right (side of ear surgery and eustachian tube dilation), *TM* Tympanic membrane, *CSOM* Chronic suppurative otitis media

Inclusion criteria were as follows: ≥ 18 years old (of both sexes); diagnosed with unilateral or bilateral persistent obstructive eustachian tube dysfunction (OETD). Exclusion criteria were the following: preoperative nasal endoscopy with evidence of anatomic conditions that would prevent transnasal access to the eustachian tube; CT temporal bone scan with evidence of carotid artery dehiscence, superior semicircular canal dehiscence, or extrinsic eustachian tube compression; patient unable to follow protocol for any reason; cleft palate or craniofacial syndrome; prior eustachian tube intervention; and prior radiation to the head and neck. The median age of patients was 37 years (range 24–77 years). All patients underwent unilateral BDET. There were 10 females and 2 males. Patient details are depicted in Table 1.

### Eustachian tube dilation (transnasal)

**Fig. 1:**
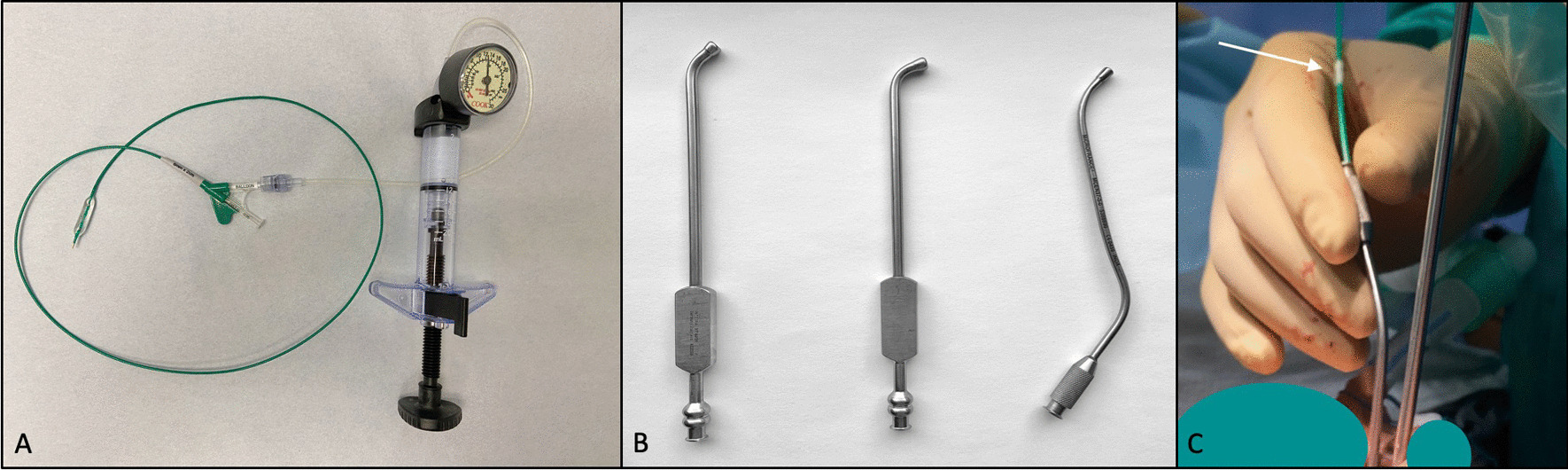
Endovascular balloon dilation system. **A** Endovascular balloon and pressure gauge. **B** Suction tips used for eustachian tube dilation. From left to right—suction tip with a 45° angled tip, suction tip with a 70° angled tip, “s” shaped malleable suction tip. **C** Balloon length marking along the distal end of endovascular balloon catheter. The EVB catheter was marked at its distal end (beyond the suction tip) to gauge the balloon length distance at which the entire balloon would be inserted into the eustachian tube

All procedures were performed under general anesthesia. BDET was carried out with an endovascular balloon (EVB: Advance^®^ 35LP, COOK Medical, Bloomington, Indiana), see Fig. 1A. The balloon is 2 cm long and 6 mm wide, dimensions [14] similar to devices approved for this specific use. Balloons were dilated to 12 atm for 2 min.

To guide the EVB to the opening of the ET, a curved metal sinus suction catheter was used (Fig. 1B). The balloon was inserted through the lumen of the suction catheter. Three different suction catheters were used. Two suction catheters were both 147 mm in length with an external diameter of 4 mm at the tip (37–14,239 Von Eicken Sinus Cannula Suction, Curved Short, Integra, Princeton, New Jersey, U.S.A.) The curved tip of said suction has an angle of about 70° (Fig. 1B); one of the suctions was altered to a 45° (Fig. 1B) angle by the manufacturer. The third suction used during the study was a malleable suction cannula (Fig. 1B), “S” shaped, 110 mm long and with a 3 mm diameter (MicroFrance^®^, Integra, Princeton, New Jersey, U.S.A.).

**Fig. 2:**
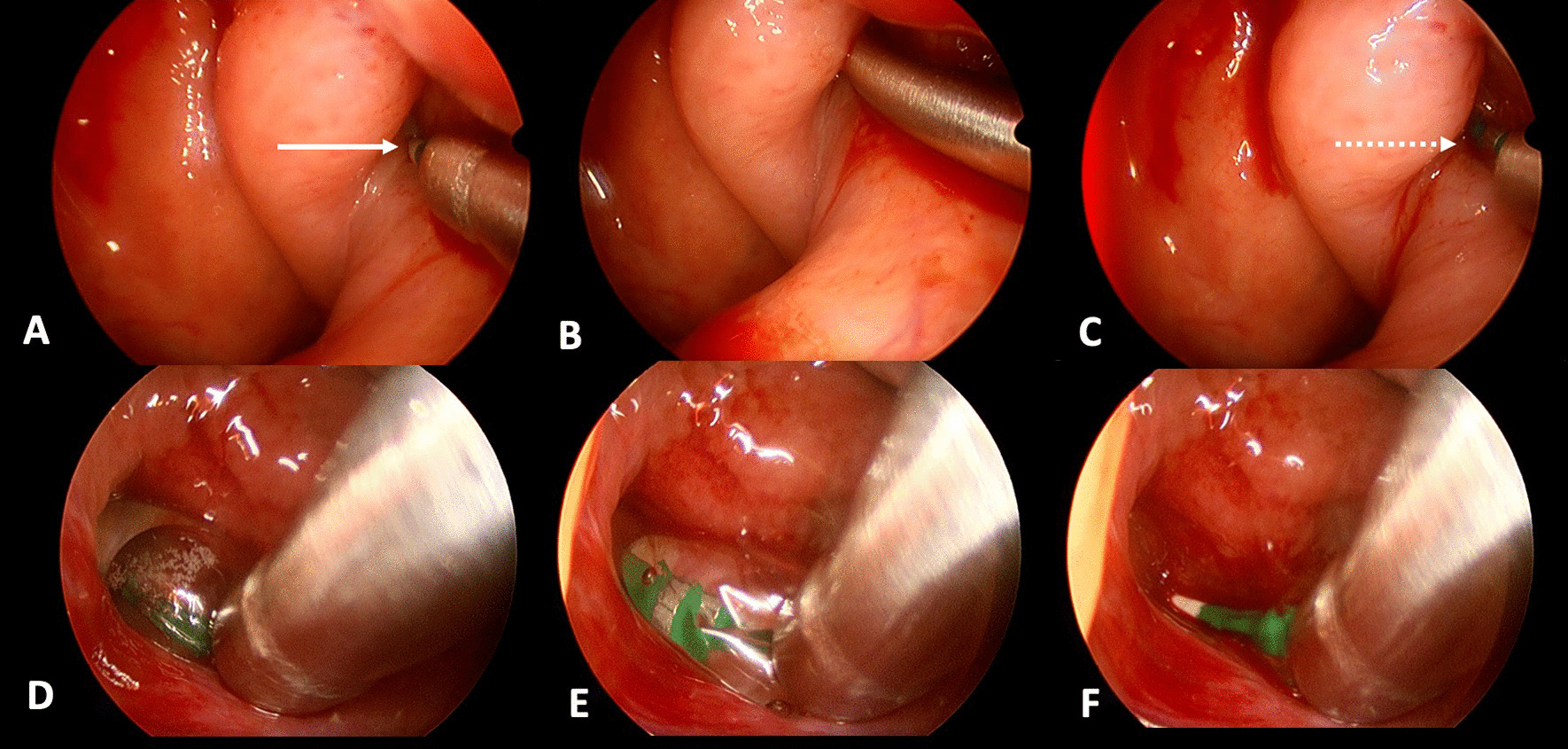
Intraoperative dilation of eustachian tube using an endovascular balloon. **A**–**C** Left orifice of the eustachian tube **A** the suction tip was brought to the orifice of the eustachian tube, the distal tip of the balloon could be visualized as white marker (solid arrow). **B** the suction tip was advanced slightly into the eustachian tube to facilitate insertion of the balloon. **C** the suction tip was retracted to visualize the distal end of the balloon—silver metal marker (dash arrow). **D**–**F** Right orifice of the eustachian tube during different steps of eustachian tube dilation. **D** the balloon was inflated to 12 atm after full insertion. **E** the balloon was deflated after 2 min. **F** the orifice is inspected after retraction of the entire system. The tip of the balloon (white) is visible

First the nasal cavity and the nasopharynx were inspected and the eustachian tube orifice identified. Once the tip of the catheter was manipulated to the ET opening, the balloon was introduced into its proximal lumen until the tip was visible at the distal end of the catheter. The EVB was inserted into the ET by slowly advancing it along the suction catheter using Seldinger technique. The suction catheter guide was then pulled back slightly to visualize the introduced balloon. The EVB was never advanced against pressure or resistance. Once the proximal silver marker of the 2 cm balloon was visible, it was not introduced further. The EVB was dilated with a pressure gauge to 12 atm. The dilated balloon was left in place for 2 min, deflated and retracted. Steps of BDET are depicted in Fig. 2. As an additional safety precaution, before inserting EVB into the ET orifice the balloon length was marked at the distal end of the suction catheter, see Fig. 1C, to avoid over-insertion of the device.

### Assessment of safety and potential damages (further outcome measures)

The orifice and the ET were inspected with a 0° and 30° endoscope to assess for any mucosal damage, adhesion, hematoma, bleeding, infection after each dilation. Photo documentation was carried out in order to review the mucosal tissue after the procedures. Further, a CT temporal bone was carried out after each surgery before the patient was discharged home. CT scan was analyzed by at least two surgeons as well as a radiologist to evaluate any potential damages. Special attention was paid to the following structures: integrity/fracture of the carotid canal, any obvious tissue changes around the ET.

### Preoperative assessment and outcome measures

Preoperatively, all patients underwent audiometry, tympanogram, nasal endoscopy, ETDQ-7 (Eustachian Tube Dysfunction Questionnaire 7) and computed tomography (CT) scan. All assessments except for ETDQ-7 are part of the standard work-up for individual patients with otologic conditions prior to surgery in the form of tympanoplasty/tympanomastoidectomy.

**Fig. 3:**
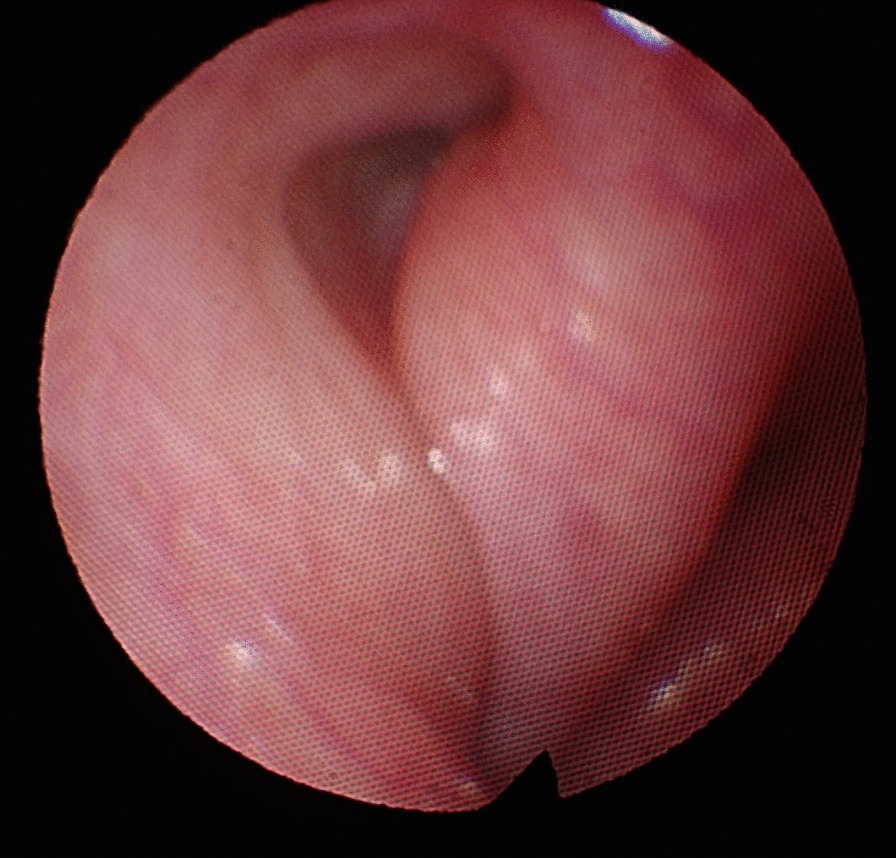
Eustachian tube orifice at the two week follow-up

Patients were seen in follow-up at two weeks and at two months after the procedure. At the two week follow up appointment, the orifice of the eustachian tube was assessed and photo-documented (Fig. 3). At two months, the ETDQ-7 was repeated as well as an audiogram, tympanogram and tympanic membrane inspection.

## Results

**Fig. 4:**
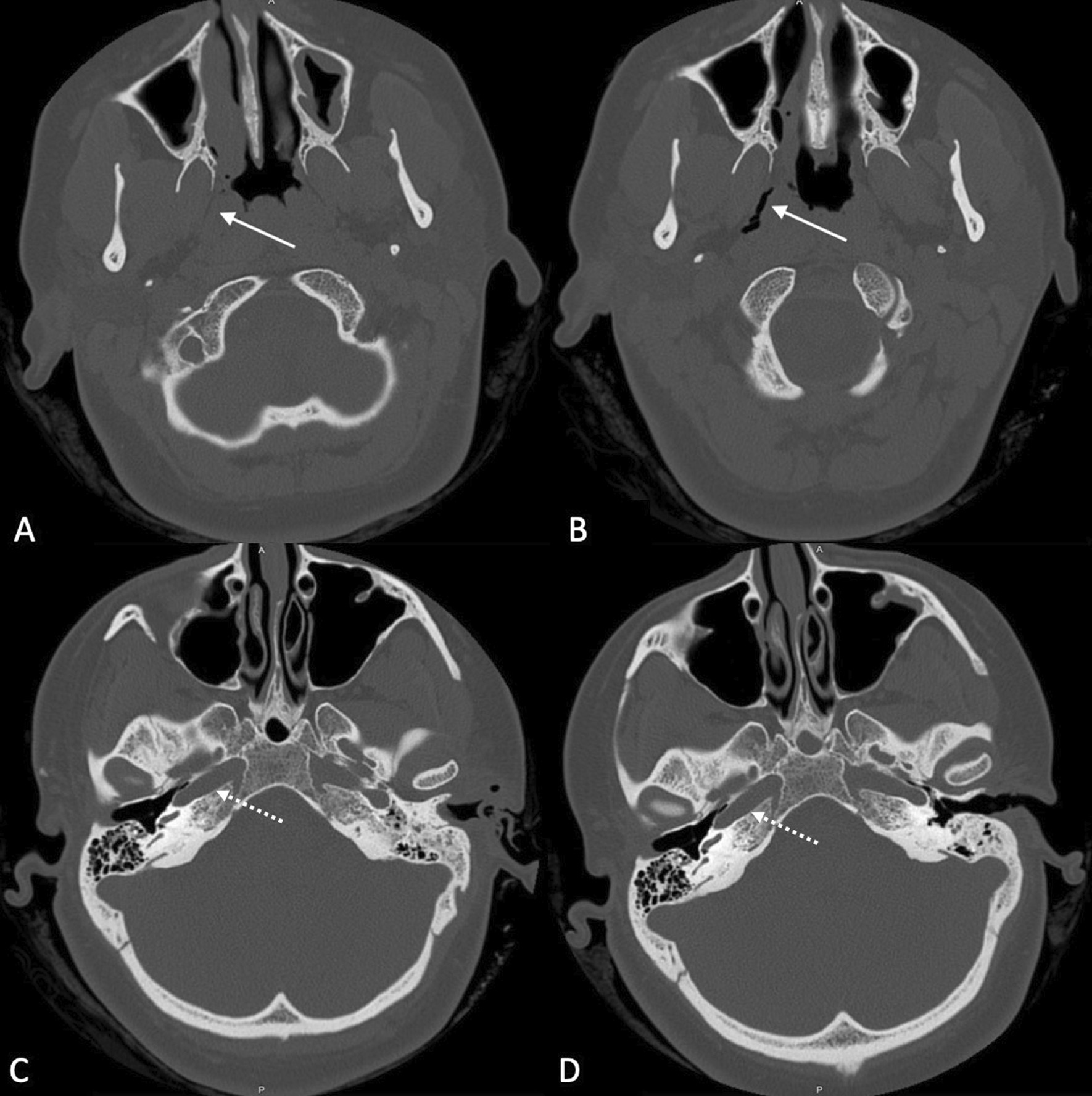
Comparison of pre- and postoperative CT (axial view). **A** preoperative CT, solid arrow points to the eustachian tube. **B** postoperative CT, solid arrow points to the dilated eustachian tube. **C** preoperative CT, dash arrow points to carotid canal before dilation. **D** postoperative CT, dash arrow points to intact carotid canal after dilation of the eustachian tube—no dehiscence can be noted

In all twelve included patients, BDET with the EVB was carried out successfully. The endoscope was placed on the same side as the dilation device in all twelve cases. The malleable suction was used in 6 cases, the 45° device in 4 cases and the 70° in 1 case. In one single case the 45° device was used first, but since placement was not successful, the malleable device was subsequently used. Although the angle of the malleable device could theoretically be bent, it was left in its original position during the entire study period. Intraoperatively minor bleeding from the turbinate was noted in some cases, which was easily controlled with a transient packing of the nose with adrenalin- or xylometazoline-soaked patties. The nasal cavity was not packed postoperatively. No instances of major adverse events occurred. The mucosa was inspected in all twelve patients after dilation and no mucosal tears or abrasions were noted. The procedure time ranged between 15 and 20 min. Postoperative CT was carried out in all twelve cases on the day of surgery to assess for any damages. Special focus was given to the tissue of the eustachian tube and any obvious damages as well as the integrity of the carotid canal in comparison to the preoperative CT. Examples are shown in Fig. 4. None of the CT images showed carotid canal fracture or dehiscence, as well as gross tissue damage. All surgeries were performed in an ambulatory day surgery setting. None of the included individuals reported any adverse events during their time at home following surgery. After two weeks of follow-up, patients were asked about the occurrence of any adverse events. One patient suffered from temporary nasal obstruction for one to two days after the procedure, which was treated with nasal saline sprays. Transnasal endoscopy did not show any swelling at follow-up. No patient reported epistaxis, swelling of the face or emphysema. Pain was not mentioned as an issue by any of the included patients. The orifice of the eustachian tube was inspected at the two-week follow-up in all cases and showed unremarkable results (Fig. 3). There was no sign of infection, bleeding, blood clot, hematoma, infection, edema at the orifice folds, torus tobarius, and nasopharynx. The mean ETDQ-7 score before the surgery was 29.5 (range 7 – 41 points), as opposed to 16 (range 7–39 points) 2 months after dilation. Results of the individual ETDQ-7 questionnaires, tympanograms, average conductive hearing loss (average across 0.5, 1, 2 and 4 kHz) and pure tone audiogram (4-PTA—average across 0.5, 1, 2 and 4 kHz) can be found in Table 1.

## Discussion

The risk profile of BDET was best highlighted by a systematic review of 15 articles (2018), which reported mild and self-limiting complications 16. In over 1800 procedures, minor adverse events occurred in 36 cases (2%). The most common adverse event was diffuse crush injury or local bleeding of the mucosa (n = 20). Additionally, hemotympanum, acute otitis media, preauricular emphysema, rhinitis, and a temporary increase in tinnitus were found in a small number of subjects.

Similarly, BDET with an endovascular balloon (EVB) in this study was both feasible and with minimal risks. Clinical effectiveness as defined by objective and subjective measures was established two months post-operatively. None of the subjects displayed worsening of symptoms or in tympanometric outcomes. Postoperative CT and endoscopic visualization of the orifice of the eustachian tube did not raise suspicion of any tissue damage or deep structural violation. There were no significant adverse events noted during the study period. Minor adverse event included temporary nasal obstruction, which occurred in one case. None of the subjects reported epistaxis, nasal pain, infection, emphysema or swelling of the face.

Technically, there are nuances that made this EVB technique more advantageous than a standard BDET balloon. First, the placement of a curved suction catheter as a conduit for the EVB insertion is facile and comes with different angles to adapt to the anatomic variations of the eustachian tubes in our subjects. The balloon tip is easy to visualize and once introduced into the orifice, does not require a guide wire for passage into the eustachian tube. We believe this feature may confer a safety advantage relative to a guidewire placement as seen in the two commercially available products currently approved for use by the US FDA and by Health Canada. Further, EVB has a wider selection of sizes and balloon profiles to allow customization. One notable difference in balloon design is the presence of a longer tapered plastic tip at the distal end of the balloon. It provides some structural rigidity to the balloon to prevent buckling as it was designed for endovascular insertion. We don’t believe this is an added risk, while marking the catheter with precise measurements to indicate balloon length to avoid over-insertion can be achieved easily both externally and internally for endoscopic visualization (Fig. 1C). The EVB catheter is much longer in its overall design, consequently, its passage will require an assistant who is also instructed to operate the injection pump. These procedural differences, we believe, confer no clinical disadvantages compared with other techniques. Dilatation can be performed multiple times without compromising balloon integrity. It’s important to advance the balloon clear of the distal end of the suction tip to avoid pinching and potentially rupturing the balloon during inflation.

The major drawback of this study is its limited sample size, the lack of a control group, and the design of a study that is not designed to establish clinical equipoise to conventional techniques. This study is a phase two clinical extension following a published cadaver study in the same vein, to establish its safety profile [14]. The current study was conducted as an adjunct to standard middle ear and mastoid procedures especially in chronic ear disease with inherent eustachian tube dysfunction. The peri-operative clinical metrics are not meant to substantiate the effectiveness of BDET using EVB in the management of eustachian tube dysfunction. Nevertheless, subjective improvement in symptoms as shown in the ETDQ-7 questionnaire, with no worsening in audiometric and tympanometric results are encouraging. Middle ears were not inspected during eustachian tube dilation, so a potential breach of the eustachian tube balloon, which has been shown for eustachian tube dilation procedures, was not inspected.

## Conclusion

We believe that the goal of this study was accomplished in confirming the safety and feasibility of BDET using an EVB as an adjunct in clinical settings. It is a necessary step and important ground-work for a larger clinical trial, currently underway, to show clinical equipoise and cost-effectiveness.

## Data Availability

Data will be made available upon reasonable request to the corresponding author.

## Abbreviations

BDET: Balloon dilation of the eustachian tube
CT: Computed tomography
ET: Eustachian tube
ETDQ-7: Eustachian tube dysfunction questionnaire 7
EVB: Endovascular balloon
FDA: Food and Drug Administration
OETD: Obstructive eustachian tube dysfunction
